# *catena*-Poly[(μ-2-amino-1,3,4-thia­diazole-κ^2^ *N* ^3^:*N* ^4^)di-μ-chlorido-cadmium]

**DOI:** 10.1107/S1600536811027048

**Published:** 2011-07-13

**Authors:** Maw-Cherng Suen, Chun-Wei Yeh, Chi-Hsiung Jou

**Affiliations:** a Department of Material and Fiber, Nanya Institute of Technology, Chung-Li 320, Taiwan; b Department of Chemistry, Chung-Yuan Christian University, Chung-Li, Taiwan; c Department of Materials and Textiles, Oriental Institute of Technology, New Taipei City, Taiwan

## Abstract

In the title coordination polymer, [CdCl_2_(C_2_H_3_N_3_S)]_*n*_, the Cd^II^ cation is coordinated by four Cl^−^ anions and two N atoms from two *trans* 2-amino-1,3,4-thia­diazole (*L*) ligands in a distorted octa­hedral geometry. The *L* ligand and Cl^−^ anions bridge adjacent Cd cations, forming a polymeric chain along the *b* axis; the separation between adjacent Cd cations is 3.619 (1) Å. In the crystal, the polymeric chains are inter­linking through N—H⋯Cl hydrogen bonds between the *L* ligands and Cl^−^ anions.

## Related literature

For background to coordination polymers, see: Kitagawa *et al.* (2004 ▶); Chiang *et al.* (2008 ▶); Yeh *et al.* (2008 ▶, 2009 ▶); Hsu *et al.* (2009 ▶). For related Cd coordination polymers, see: Suen & Wang (2007*a* ▶,*b* ▶).
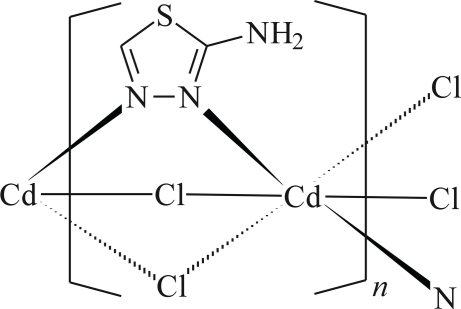


## Experimental

### 

#### Crystal data

[CdCl_2_(C_2_H_3_N_3_S)]
*M* *_r_* = 284.43
Monoclinic, 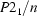

*a* = 7.7264 (6) Å
*b* = 7.2227 (6) Å
*c* = 12.7608 (11) Å
β = 95.489 (2)°
*V* = 708.86 (10) Å^3^
*Z* = 4
Mo *K*α radiation
μ = 4.04 mm^−1^
*T* = 297 K
0.48 × 0.46 × 0.34 mm

#### Data collection

Bruker APEXII CCD diffractometer
Absorption correction: multi-scan (*SADABS*; Bruker, 2000 ▶) *T*_min_ = 0.170, *T*_max_ = 0.341
3718 measured reflections
1381 independent reflections
1354 reflections with *I* > 2σ(*I*)
*R*_int_ = 0.019

#### Refinement

*R*[*F* ^2^ > 2σ(*F* ^2^)] = 0.021
*wR*(*F* ^2^) = 0.056
*S* = 1.16
1381 reflections
83 parameters
1 restraint
H-atom parameters constrained
Δρ_max_ = 0.61 e Å^−3^
Δρ_min_ = −0.76 e Å^−3^

### 

Data collection: *APEX2* (Bruker, 2010 ▶); cell refinement: *SAINT* (Bruker, 2010 ▶); data reduction: *SAINT*; program(s) used to solve structure: *SHELXS97* (Sheldrick, 2008 ▶); program(s) used to refine structure: *SHELXL97* (Sheldrick, 2008 ▶); molecular graphics: DAIMOND (Brandenburg, 2010 ▶); software used to prepare material for publication: *SHELXL97*.

## Supplementary Material

Crystal structure: contains datablock(s) I, global. DOI: 10.1107/S1600536811027048/xu5265sup1.cif

Structure factors: contains datablock(s) I. DOI: 10.1107/S1600536811027048/xu5265Isup2.hkl

Additional supplementary materials:  crystallographic information; 3D view; checkCIF report

## Figures and Tables

**Table 1:** Selected bond lengths (Å)

Cd—N1	2.361 (2)
Cd—N2^i^	2.341 (2)
Cd—Cl1	2.6262 (7)
Cd—Cl1^ii^	2.6697 (7)
Cd—Cl2	2.6583 (7)
Cd—Cl2^ii^	2.6222 (7)

Symmetry codes: (i) 
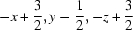
; (ii) 
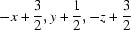
.

**Table 2:** Hydrogen-bond geometry (Å, °)

*D*—H⋯*A*	*D*—H	H⋯*A*	*D*⋯*A*	*D*—H⋯*A*
N3—H3*A*⋯Cl2^iii^	0.86	2.60	3.390 (3)	154
N3—H3*B*⋯Cl2^iv^	0.86	2.77	3.216 (3)	114

Symmetry codes: (iii) 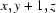; (iv) 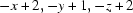.

**Fig. 1.**
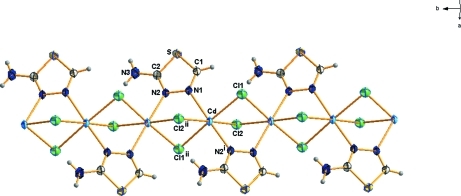
A portion of the one-dimensional chain. Ellipsoids are drawn at 30% probability level, and H atoms of spheres of arbitrary radius. Symmetry codes: (i) -x + 3/2, y - 1/2, -z + 3/2; (ii) -x + 3/2, y + 1/2, -z + 3/2.

**Fig. 2.**
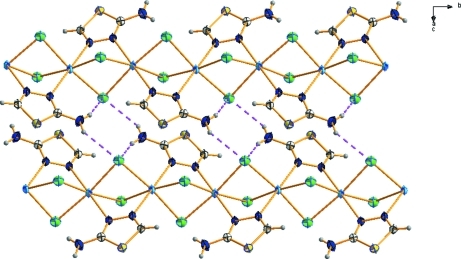
The packing diagram shows the N—H···Cl hydrogen bonds among the one-dimensional Chains.

## supplementary crystallographic information

### Comment

The synthesis of metal coordination polymers has been a subject of intense research due to their interesting structural chemistry and potential applications in gas storage, separation, catalysis, magnetism, luminescence, and drug delivery (Kitagawa *et al.*, 2004). Roles of anion, solvent and ligand comformations in self-assembly of coordination complexes containing polydentate nitrogen ligands are very intersting (Chiang *et al.*, 2008; Yeh *et al.*, 2008; Hsu *et al.*, 2009; Yeh *et al.*, 2009). Tha Cd(II) complexes containing polydentate ligands showing various type frameworks are also reported (Suen & Wang, 2007*a*,*b*). The Cd^2+^ cations are six-coordinate, which are coordinated with four Cl atoms and two N atoms from two *L* ligands (Fig. 1). The Cd···Cd distance separated by the bridging *L* ligands and Cl atoms is 10.257 (1) and 3.619 (1) Å. The one-dimensional polymeric chains are interlinking through N—H···Cl hydrogen bonds between the *L* ligands and Cl anions in the crystal structure (Fig. 2, Tab.1).

### Experimental

An aqueous solution (5.0 ml) of cadmium chloride (1.0 mmol) was layered carefully over a methanolic solution (5.0 ml) of 2-amino-1,3,4-thiadiazole (1.0 mmol) in a tube. Colourless crystals were obtained after several weeks. These were washed with methanol and collected in 68.7% yield.

### Refinement

H atoms were contrained to ideal geometries with C—H = 0.93 and N—H = 0.86 Å and *U*_iso_(H) = 1.2*U*_eq_(C,N).

### Crystal data

**Table:**

[CdCl_2_(C_2_H_3_N_3_S)]	*F*(000) = 536
*M**_r_* = 284.43	*D*_x_ = 2.665 Mg m^−^^3^
Monoclinic, *P*2_1_/*n*	Mo *K*α radiation, λ = 0.71073 Å
Hall symbol: -P 2yn	Cell parameters from 3467 reflections
*a* = 7.7264 (6) Å	θ = 2.7–26.0°
*b* = 7.2227 (6) Å	µ = 4.04 mm^−^^1^
*c* = 12.7608 (11) Å	*T* = 297 K
β = 95.489 (2)°	Parallelepiped, colourless
*V* = 708.86 (10) Å^3^	0.48 × 0.46 × 0.34 mm
*Z* = 4	

### Data collection

**Table:**

Bruker APEXII CCD diffractometer	1381 independent reflections
Radiation source: fine-focus sealed tube	1354 reflections with *I* > 2σ(*I*)
graphite	*R*_int_ = 0.019
φ and ω scans	θ_max_ = 26.0°, θ_min_ = 3.0°
Absorption correction: multi-scan (*SADABS*; Bruker, 2000)	*h* = −9→9
*T*_min_ = 0.170, *T*_max_ = 0.341	*k* = −8→7
3718 measured reflections	*l* = −15→10

### Refinement

**Table:**

Refinement on *F*^2^	Secondary atom site location: difference Fourier map
Least-squares matrix: full	Hydrogen site location: inferred from neighbouring sites
*R*[*F*^2^ > 2σ(*F*^2^)] = 0.021	H-atom parameters constrained
*wR*(*F*^2^) = 0.056	*w* = 1/[σ^2^(*F*_o_^2^) + (0.0313*P*)^2^ + 0.7123*P*] where *P* = (*F*_o_^2^ + 2*F*_c_^2^)/3
*S* = 1.16	(Δ/σ)_max_ = 0.001
1381 reflections	Δρ_max_ = 0.61 e Å^−^^3^
83 parameters	Δρ_min_ = −0.76 e Å^−^^3^
1 restraint	Extinction correction: *SHELXL97* (Sheldrick, 2008), Fc^*^=kFc[1+0.001xFc^2^λ^3^/sin(2θ)]^-1/4^
Primary atom site location: structure-invariant direct methods	Extinction coefficient: 0.0097 (6)

### Special details

**Table:**

Experimental. Refinement of *F*^2^ against ALL reflections. The weighted *R*-factor *wR* and goodness of fit *S* are based on *F*^2^, conventional *R*-factors *R* are based on *F*, with *F* set to zero for negative *F*^2^. The threshold expression of *F*^2^ > σ(*F*^2^) is used only for calculating *R*-factors(gt) *etc*. and is not relevant to the choice of reflections for refinement. *R*-factors based on *F*^2^ are statistically about twice as large as those based on *F*, and *R*- factors based on ALL data will be even larger.
Geometry. All e.s.d.'s (except the e.s.d. in the dihedral angle between two l.s. planes) are estimated using the full covariance matrix. The cell e.s.d.'s are taken into account individually in the estimation of e.s.d.'s in distances, angles and torsion angles; correlations between e.s.d.'s in cell parameters are only used when they are defined by crystal symmetry. An approximate (isotropic) treatment of cell e.s.d.'s is used for estimating e.s.d.'s involving l.s. planes.
Refinement. Refinement of *F*^2^ against ALL reflections. The weighted *R*-factor *wR* and goodness of fit *S* are based on *F*^2^, conventional *R*-factors *R* are based on *F*, with *F* set to zero for negative *F*^2^. The threshold expression of *F*^2^ > σ(*F*^2^) is used only for calculating *R*-factors(gt) *etc*. and is not relevant to the choice of reflections for refinement. *R*-factors based on *F*^2^ are statistically about twice as large as those based on *F*, and *R*- factors based on ALL data will be even larger.

### Fractional atomic coordinates and isotropic or equivalent isotropic displacement parameters (Å^2^)

**Table:**

	*x*	*y*	*z*	*U*_iso_*/*U*_eq_	
Cd	0.73571 (3)	0.17322 (3)	0.745512 (14)	0.02441 (12)	
Cl1	0.51717 (10)	−0.08486 (9)	0.79344 (6)	0.02966 (18)	
Cl2	0.97270 (10)	−0.06290 (9)	0.83088 (6)	0.03076 (18)	
S	0.70167 (11)	0.40964 (10)	1.09911 (6)	0.03228 (19)	
N1	0.7182 (3)	0.3292 (3)	0.90672 (19)	0.0253 (5)	
N2	0.7623 (3)	0.5146 (3)	0.91436 (18)	0.0245 (5)	
N3	0.7988 (4)	0.7503 (4)	1.0408 (2)	0.0412 (7)	
H3A	0.8242	0.8302	0.9947	0.049*	
H3B	0.7969	0.7827	1.1056	0.049*	
C1	0.6831 (4)	0.2586 (4)	0.9949 (2)	0.0281 (6)	
H1A	0.6504	0.1356	1.0016	0.034*	
C2	0.7623 (4)	0.5757 (4)	1.0118 (2)	0.0260 (6)	

### Atomic displacement parameters (Å^2^)

**Table:**

	*U*^11^	*U*^22^	*U*^33^	*U*^12^	*U*^13^	*U*^23^
Cd	0.03477 (16)	0.01300 (14)	0.02532 (16)	0.00063 (7)	0.00221 (9)	−0.00127 (6)
Cl1	0.0319 (4)	0.0198 (3)	0.0383 (4)	−0.0011 (3)	0.0083 (3)	0.0025 (3)
Cl2	0.0327 (4)	0.0180 (3)	0.0395 (4)	−0.0019 (3)	−0.0070 (3)	0.0008 (3)
S	0.0457 (5)	0.0284 (4)	0.0229 (4)	−0.0039 (3)	0.0040 (3)	0.0012 (3)
N1	0.0344 (13)	0.0146 (11)	0.0270 (12)	0.0000 (9)	0.0037 (10)	−0.0012 (9)
N2	0.0324 (12)	0.0156 (11)	0.0255 (11)	−0.0012 (9)	0.0025 (9)	−0.0007 (9)
N3	0.0633 (19)	0.0271 (14)	0.0331 (14)	−0.0125 (13)	0.0037 (13)	−0.0069 (11)
C1	0.0370 (16)	0.0189 (14)	0.0282 (14)	−0.0014 (12)	0.0027 (11)	0.0014 (11)
C2	0.0298 (15)	0.0218 (14)	0.0258 (14)	0.0001 (11)	−0.0006 (11)	−0.0006 (10)

### Geometric parameters (Å, °)

**Table:**

Cd—N1	2.361 (2)	N1—C1	1.287 (4)
Cd—N2^i^	2.341 (2)	N1—N2	1.383 (3)
Cd—Cl1	2.6262 (7)	N2—C2	1.320 (4)
Cd—Cl1^ii^	2.6697 (7)	N2—Cd^ii^	2.341 (2)
Cd—Cl2	2.6583 (7)	N3—C2	1.337 (4)
Cd—Cl2^ii^	2.6222 (7)	N3—H3A	0.8600
S—C1	1.715 (3)	N3—H3B	0.8600
S—C2	1.731 (3)	C1—H1A	0.9300
			
N2^i^—Cd—N1	177.00 (9)	C1—S—C2	87.11 (14)
N2^i^—Cd—Cl2^ii^	94.97 (6)	C1—N1—N2	113.1 (2)
N1—Cd—Cl2^ii^	83.84 (6)	C1—N1—Cd	127.38 (19)
N2^i^—Cd—Cl1	85.04 (6)	N2—N1—Cd	119.28 (16)
N1—Cd—Cl1	92.52 (6)	C2—N2—N1	111.6 (2)
Cl2^ii^—Cd—Cl1	102.52 (2)	C2—N2—Cd^ii^	131.18 (19)
N2^i^—Cd—Cl2	88.94 (6)	N1—N2—Cd^ii^	115.76 (16)
N1—Cd—Cl2	92.51 (6)	C2—N3—H3A	120.0
Cl2^ii^—Cd—Cl2	173.27 (2)	C2—N3—H3B	120.0
Cl1—Cd—Cl2	83.24 (2)	H3A—N3—H3B	120.0
N2^i^—Cd—Cl1^ii^	95.37 (6)	N1—C1—S	114.6 (2)
N1—Cd—Cl1^ii^	87.23 (6)	N1—C1—H1A	122.7
Cl2^ii^—Cd—Cl1^ii^	83.09 (2)	S—C1—H1A	122.7
Cl1—Cd—Cl1^ii^	174.324 (18)	N2—C2—N3	123.8 (3)
Cl2—Cd—Cl1^ii^	91.11 (2)	N2—C2—S	113.5 (2)
Cd—Cl1—Cd^i^	86.22 (2)	N3—C2—S	122.6 (2)
Cd^i^—Cl2—Cd	86.53 (2)		

Symmetry codes: (i) −*x*+3/2, *y*−1/2, −*z*+3/2; (ii) −*x*+3/2, *y*+1/2, −*z*+3/2.

### Hydrogen-bond geometry (Å, °)

**Table:**

*D*—H···*A*	*D*—H	H···*A*	*D*···*A*	*D*—H···*A*
N3—H3A···Cl2^iii^	0.86	2.60	3.390 (3)	154
N3—H3B···Cl2^iv^	0.86	2.77	3.216 (3)	114

Symmetry codes: (iii) *x*, *y*+1, *z*; (iv) −*x*+2, −*y*+1, −*z*+2.
